# Healthcare costs of COVID-19 hospitalization in maintenance hemodialysis patients during the first two pandemic waves in Romania: a retrospective cohort study

**DOI:** 10.25122/jml-2026-0056

**Published:** 2026-04

**Authors:** Oana Nicolescu, Mihaela Magdalena Mitache, Andrei Mitache, Adelina-Gabriela Niculescu, Dragos Garofil, Victor Dan Eugen Strambu, Bogdan Oancea, Marian Necula, Corneliu Ovidiu Vrancianu, Ioana Ruxandra Poiana, Adrian Radu Petru, Ana Maria Alexandra Stănescu

**Affiliations:** 1 Faculty of Medicine, Carol Davila University of Medicine and Pharmacy, Bucharest, Romania; 2 Faculty of Medicine, Titu Maiorescu University, Bucharest, Romania; 3 Public Health Directorate, Bucharest, Romania; 4 Research Institute of the University of Bucharest (ICUB), University of Bucharest, Bucharest, Romania; 5 Department of Science and Engineering of Oxide Materials and Nanomaterials, National University of Science and Technology, Politehnica, Bucharest, Romania; 6 Dr. Carol Davila Clinical Hospital of Nephrology, Bucharest, Romania; 7 National Institute of Research and Development for Biological Sciences, Bucharest, Romania; 8 Faculty of Administration and Business, University of Bucharest, Bucharest, Romania; 9 Doctoral School, Carol Davila University of Medicine and Pharmacy, Bucharest, Romania; 10 Academy of Romanian Scientists (AOSR), Bucharest, Romania

**Keywords:** COVID-19, hemodialysis, chronic kidney disease, hospitalization cost, healthcare expenditure, Romania

## Abstract

Patients receiving maintenance hemodialysis were highly vulnerable during the coronavirus disease 2019 pandemic because treatment continuity required repeated exposure to healthcare settings, while multiple comorbidities increased severe infection risk. Although many studies reported clinical outcomes in dialysis populations, fewer assessed hospitalization costs, particularly in Eastern Europe. This study evaluated hospitalization costs among maintenance hemodialysis patients admitted with COVID-19 during pandemic waves in Romania: Wave 1 (March–May 2020), Wave 2 (October 2020–February 2021), and Wave 3 (July–November 2021). We conducted a retrospective cohort study using linked administrative and clinical databases from a Romanian tertiary nephrology center. The source database included 126 entries. After exclusion of records lacking complete reimbursement or admission/discharge data, 84 hospitalization episodes were eligible for analysis. The dataset included the first two waves: 23 admissions in Wave 1 and 61 in Wave 2. Mean hospitalization cost increased from 6,216.51 RON in Wave 1 to 8,478.76 RON in Wave 2, while median cost increased from 3,094.44 RON to 5,281.90 RON. Mean length of stay decreased significantly from 18.39 to 11.61 days (*P* = 0.0047). Mean cost per hospitalization day increased significantly from 426.30 RON/day to 767.47 RON/day (*P* = 0.0013). Total hospitalization cost correlated positively with length of stay (Spearman’s ρ = 0.50, *P* < 0.001). Data from Wave 3 were limited and interpreted descriptively. The second pandemic wave was associated with shorter admissions and higher daily hospitalization costs, suggesting more intensive inpatient management. These findings support prioritization of dialysis patients in future preparedness and healthcare resource planning.

## Introduction

The COVID-19 pandemic generated one of the greatest recent challenges for healthcare systems worldwide. Recurrent waves of infection were associated with increased hospital admissions, high demand for critical care beds, staff shortages, and substantial treatment expenditure [1,2]. In many countries, hospitals were required to reorganize clinical pathways rapidly while maintaining continuity of care for patients with chronic diseases.

Among vulnerable chronic disease populations, patients receiving maintenance hemodialysis represented a particularly high-risk group. Unlike many outpatient treatments, dialysis cannot usually be interrupted or postponed. Patients must attend healthcare facilities several times weekly, which limits strict isolation and increases exposure during periods of community transmission [3,4]. In addition, dialysis patients frequently present with advanced age, cardiovascular disease, diabetes mellitus, chronic inflammation, frailty, and impaired immune response, all of which may worsen outcomes after severe acute respiratory syndrome coronavirus 2 (SARS-CoV-2) infection [5,6].

Early reports from Europe, North America, and multinational dialysis registries described increased hospitalization and mortality rates in hemodialysis patients with COVID-19 compared with the general population [7-10]. Beyond clinical risk, this population also presents major logistical challenges during hospitalization. Renal replacement therapy must continue during acute illness, requiring dialysis scheduling, infection control measures, vascular access surveillance, fluid balance management, electrolyte correction, and multidisciplinary care involving nephrology, internal medicine, infectious disease, and, at times, intensive care teams [4,11].

The economic consequences of COVID-19 hospitalization have been described in general medical populations, where prolonged admissions, respiratory support, repeated investigations, and critical care utilization substantially increased direct medical costs [12,13]. However, specific economic data focused on maintenance hemodialysis patients remains limited, especially in Eastern Europe. Such information may be useful for hospital administrators, clinicians, and public health decision-makers when planning future responses to infectious outbreaks.

Romania experienced several pandemic waves with changing epidemiological dynamics and increasing healthcare pressure. National epidemiological investigations, including the first Romanian seroprevalence survey reported by Pistol *et al*., documented progressive population exposure to SARS-CoV-2 during the early pandemic period [13]. The present study aimed to assess hospitalization costs among maintenance hemodialysis patients admitted with COVID-19 during the first two Romanian pandemic waves, using complete administrative records from a tertiary nephrology center.

## Material and Methods

### Study design

We conducted a retrospective cohort study using anonymized administrative and clinical data from a Romanian tertiary nephrology hospital that managed patients hospitalized with COVID-19.

### Data source and eligibility criteria

The source database contained 126 entries. Records were reviewed for completeness of three essential variables: reimbursement value, admission date, and discharge date. Entries lacking complete information were excluded from the final economic analysis. After data cleaning, 84 hospitalization episodes remained eligible.

### Pandemic wave classification

Eligible admissions were grouped according to predefined national pandemic periods. Wave 1 included hospitalizations from March 2020 to May 2020, whereas Wave 2 included hospitalizations from October 2020 to February 2021. Later periods were not included because sufficiently complete records were unavailable for robust comparative analysis.

### Outcomes

The primary outcome was total hospitalization cost, expressed in Romanian lei (RON). Secondary outcomes included length of stay, cost per hospitalization day, and correlation between hospitalization cost and length of stay.

### Statistical analysis

Continuous variables were summarized as mean ± standard deviation, median, interquartile range, and minimum and maximum values, as appropriate. Because reimbursement values showed a non-normal distribution, comparisons between groups were performed using the Mann–Whitney U test. Correlations between continuous variables were assessed using Spearman’s rank correlation coefficient. A *P* value below 0.05 was considered statistically significant. Statistical analyses were interpreted as exploratory due to the retrospective design and limited sample size.

## Results

### Study population

Among the 84 eligible hospitalization episodes, 23 occurred during Wave 1 and 61 during Wave 2.

### Hospitalization cost

Mean hospitalization cost was 6,216.51 RON during Wave 1 and 8,478.76 RON during Wave 2. Median values were 3,094.44 RON and 5,281.90 RON, respectively. Although average expenditure was higher during the second wave, the difference did not reach statistical significance (*P* = 0.405).

### Length of stay

Mean length of stay decreased significantly from 18.39 days during Wave 1 to 11.61 days during Wave 2 (*P* = 0.0047). Median length of stay decreased from 15 to 10 days.

### Cost per hospitalization day

Mean daily hospitalization cost increased significantly from 426.30 RON/day during Wave 1 to 767.47 RON/day during Wave 2 (*P* = 0.0013).

### Correlation between cost and hospitalization duration

Across the entire cohort, total hospitalization costs correlated positively with length of stay (Spearman’s ρ = 0.50, *P* < 0.001), indicating that longer admissions were generally associated with higher expenditure (Table 1).

**Table 1:** Comparative economic indicators according to pandemic wave

Variable	Wave 1 (*n* = 23)	Wave 2 (*n* = 61)	*P value*
Mean cost (RON)	6,216.51	8,478.76	0.405
Median cost (RON)	3,094.44	5,281.90	—
Mean length of stay (days)	18.39	11.61	0.0047
Median length of stay (days)	15	10	—
Mean cost/day (RON)	426.30	767.47	0.0013

### High-cost admissions

Several hospitalizations resulted in markedly elevated reimbursement amounts, exceeding 28,000 RON. These episodes likely reflected prolonged hospitalization, increased treatment intensity, or multiple complications requiring additional care.

## Discussion

The present study showed that maintenance hemodialysis patients hospitalized with COVID-19 generated substantial healthcare expenditure during the first two Romanian pandemic waves. The most relevant finding was not a statistically significant increase in total cost per admission, but rather a significant shift in the cost structure between the two periods.

During the second wave, admissions were shorter, while daily hospitalization costs were significantly higher. This pattern suggests more concentrated resource use over shorter hospital stays. Several clinically plausible explanations may account for this observation.

First, inpatient management had become more standardized by the second pandemic wave. Clinical pathways increasingly incorporate repeated laboratory monitoring, chest imaging, thromboprophylaxis, corticosteroids, oxygen therapy, and closer surveillance for respiratory deterioration [14,15]. Such interventions may increase daily expenditure even when hospitalization duration declines.

Second, dialysis patients require resource-intensive background care independent of COVID-19 severity. Renal replacement therapy must continue throughout admission, while clinicians must also manage vascular access, fluid balance, electrolyte disturbances, anemia, and cardiovascular instability. Consequently, hospitalization of dialysis-dependent patients may require substantially more resources than standard medical admissions [4,11].

Third, shorter admissions during the second wave may reflect improved clinical organization, greater physician experience, faster triage pathways, and pressure to optimize bed turnover during periods of increased demand. Similar adaptive patterns were reported internationally as healthcare systems adjusted to successive pandemic phases [2,16]. Comparable Romanian evidence was later reported by Popescu *et al*., who identified substantial ICU-associated costs during a subsequent pandemic wave in an Eastern European tertiary hospital [17].

Comparable reorganizations of hospital activity were also reported in Romania, where Garofil *et al*. described substantial service adaptations during the pandemic period [14].

The positive correlation between total cost and length of stay was expected, since prolonged hospitalization generally increases cumulative expenditure. However, the significant increase in daily cost indicates that treatment intensity also contributed materially to the overall financial burden.

Our findings are consistent with broader COVID-19 cost-of-illness studies showing that inpatient expenditure rises substantially when hospitalization requires advanced monitoring, respiratory support, or prolonged treatment [12,13]. However, the present study adds dialysis-specific data from Romania, a region where published economic evidence remains scarce.

From a practical perspective, these results support continued prioritization of dialysis patients in emergency preparedness strategies. During future outbreaks, maintaining dialysis continuity, reducing nosocomial transmission, ensuring early diagnosis, and preventing severe disease may reduce both clinical complications and hospital expenditure.

### Limitations

This study has several limitations. First, it was retrospective and based on administrative reimbursement data, which may not capture all direct and indirect costs. Second, the analysis was conducted in a single tertiary center, limiting generalizability. Third, detailed patient-level clinical severity variables were not consistently available for all admissions. Fourth, later pandemic waves could not be analyzed comparatively because sufficiently complete records were unavailable.

## Conclusion

After restricting the analysis to complete hospitalization records, the second Romanian COVID-19 wave was associated with significantly shorter admissions and significantly higher daily hospitalization costs in maintenance hemodialysis patients. These findings suggest more intensive inpatient management during later pandemic phases and support prioritization of dialysis patients in future healthcare preparedness, vaccination strategies, and resource allocation planning, in line with the importance of protective immune responses highlighted in previous Romanian studies.

## Data Availability

The original contributions presented in this study are included in the article. Further inquiries can be directed to the corresponding author.

## Personal thanks

We acknowledge the UEFISCDI project PN-IV-P6-6.1-CoEx-2024-0196, Acronym ARES, Contract no. 17/2.03.2026, and the Ministry of Investments and European Projects, through the Managing Authority for the Health Program, PS/272/PS_P5/OP1/RSO1.1/PS_P5_RSO1.1_A9—ROGEN Project (MySMIS 324809). During the preparation of this manuscript, the authors used ChatGPT Plus (OpenAI, GPT-5.2, December 2025 version) to improve clarity, grammar, and logical flow in the English-language sections. The authors have reviewed and edited the output and take full responsibility for the content of this publication.
